# Evaluation of Toll-Like Receptor 11 Agonist Adjuvant Activity in Immunization of BALB/c Mice with Total Lysate Antigens of *Toxoplasma gondii* RH Strain

**DOI:** 10.18502/ijpa.v15i3.4199

**Published:** 2020

**Authors:** Meysam SHOKRI, Khosro HAZRATI TAPPEH, Elyar MESHKINI, Arash AMINPOUR

**Affiliations:** Department of Parasitology and Mycology, School of Medicine, Urmia University of Medical Sciences, Urmia, Iran

**Keywords:** *Toxoplasma gondii*, Adjuvant, Toll-like receptor 11, Immunization, *Toxoplasma* lysate antigen

## Abstract

**Background:**

In this study, the effect of total lysate antigen (TLA) of *Toxoplasma gondii* on spleen lymphocyte prolifration, secretion of IL5, INF-γ, and mice survival time was evaluated using agonist of toll-like receptor (TLR) 11, as an adjuvant.

**Methods:**

This study was done in the Department of Parasitology and Mycology of Urmia University of Medical Sciences, Urmia, Iran in 2018. First, different groups of BALB/c mice were immunized with TLA alone and also TLA + TLR11 agonist. Subsequently, factors such as spleen lymphocyte prolifration, IL-5, and INF-γ secretion and mice survival time in different groups were compared.

**Results:**

Mice immunized with TLA + adjuvant showed higher immunization index than the two other groups and combination of TLR11 (as an adjuvant) and TLA significantly elevated the effect of TLA by increasing the production of INF-γ and IL-5 and by the shift of the immune system to Th1. In addition, the combination of TLA and TLR11 adjuvant increased the proliferation of lymphocytes and survival time in mice against *T. gondii.*

**Conclusion:**

Profilin (as an adjuvant) in combination with TLA could be a potent vaccine candidate that evokes a powerful specific immune response and significantly improves the efficacy of TLA vaccine by increasing the induction of INF-γ production and by shifting the immune responses to Th1 profile through increasing the INF-γ/IL-5 ratio. It causes significant protection against *T. gondii* after i.p. injection.

## Introduction

*Toxoplasma gondii* is an intracellular protozoa and the causative agent of toxoplasmosis. It is a common parasite between humans and animals and is responsible for the infection of most vertebrates of warm-blooded animals. Based on a previous serology study, about one-third of populations in most countries are infected by this parasite (1). The majority of chronic toxoplasmosis is asymptomatic, and the most common signs in acute toxoplasmosis are mainly lymphadenopaty (2). In congenital toxoplasmosis, if a mother is exposed to *T. gondii* for the first time during her pregnancy period, it may cause serious damages to the fetus such as central nervous system failure, spontaneous abortion, and fetal death, and a set of symptoms such as rash, fever, liver enlargement, jaundice, hydrocephalus, microcephaly, and retinochoroiditis (3). In immunocompromised individuals (e.g. patients with HIV/AIDS), patients with cancer, and transplant recipients, infection with this oportunistic pathogen can be fatal (4).

Immunity to toxoplasmosis emerges after an acute stage, and the formation of a chronic phase persists as long as the infectious agent remains in the body. Both humoral and cellular immune responses play a significant role in immunity. In humoral immunity, inflammation produces cytokines IL-4, IL-5, IL-10, and IL-12 stimulated by TH2 cells. It increases the number of eosinophils and their cytotoxic activity with further stimulation of degranulation and provides long-term protection by eosinophilia. In cellular immunity, the parasite enters the macrophages by binding to collagen and laminin receptors on the surface of the macrophages, produces peneterating-enhancing factor and then duplicates. Finally, the infected macrophage produces IL-12, which increases the number of T helper 1 (Th1) cells. Th1 cells, in turn, produce interferon-gamma (IFN-γ), which activates CD8+ cells and destroys the infected macrophages. Therefore, in toxoplasmosis, cellular immunity is more important than humoral immunity (5–7). In addition, INF-γ is essential for the induction of T cell responses and resistance to *T. gondii* (8).

Advances in the production of a powerful vaccine against toxoplasma has revealed that adjuvants are pivotal players in the induction of immune system for eliminating this parasite. The term ‘adjuvant’ is originally derived from the latin word adjutants meaning ‘ancillary’. Adjuvants are categorized into three main groups: (I) active immunostimulants, (II) transport proteins that provide T cell help, and (III) vehicle adjuvants such as materials that serve as a matrix for antigens and stimulate the immune response (9). In toxoplasma infection, they indirectly activate innate immunity through affecting pattern recognition receptors or directly function as cytokines. Innate immunity cells express a range of pattern recognition receivers that are capable of infecting infectious agents; one of known as the toll-like receptor (TLR) (10). TLR2, due to its association with TLR1 or TLR6, can recognize lipomannan from mycobacterium (11–17), the lipoteichoic acid from Gram-positive bacteria and diacylated and tryacylated bacterial lipopeptides, as well as glycosylphosphatidylinositol anchor structures from *Trypanosoma cruzi* (18). TLR1-TLR13 can have an impact on the signaling pathway of TLR receptors as adjuvants. The TLR11 agonist, known as flagellin, serves as one of the most important adjuvants of the immune system. Flagellin is capable of detecting specific ligands of *T. gondii*, thereby stimulating the Th1 cellular immune system (19–30).

Profilins are a class of small actin-binding proteins present only in the eukaryotic cells and have a regulatory role in the polymerization of actin. Profilin is released from dentritic cells and is a very powerful stimulator of IL-12. It upregulates the inflamatory modulators such as MCP-1, IL-6, TNF-α, and INF-γ and has antitumor properties. The antiviral properties of profilin has demonstrated that when it is administrated to mice, alone or in combination with agonist, resistance against acute phlebovirus occurs (31). Profilin is a molecule secreted by *T. gondii*, activates TLR11, manages parasite motility, and hosts cell invasion and has an important role in Th1 cells responses, to prevent *T. gondii* infection in a murine model (32).

In this study, we evaluated the total tachyzoite antigens of *T. gondii* in combination with the TLR11 (profilin) agonist as an adjuvant in inducing homoral and cellular immunity and also assessed its efficiency in protecting BALB/c mice against the virulent RH strain of *T. gondii*.

## Materials and Methods

### Mice

This study was done in 2018. Six- to eight-week-old inbred female BALB/c mice were purchased from Razi Vaccine and Serum Research Institute (Karaj, Iran). All mice were documented to be specific pathogen-free and to receive a diet of commercial food pellet and water *ad libitum*.

All experiments were conducted following the protocol approved by the Institutional Animal Care and Use Committee at Urmia University of Medical Sciences (Urmia, Iran).

### Toxoplasma lysate antigaen (TLA) prepartion

The tachyzoites of *T. gondii* RH strain donated by Institut Pasteur (Paris, France) were repeatedly injected into the peritoneum of mice. Parasites in the peritoneal fluid were collected after centrifugation and washed three times with sterile 1× phosphate-buffered saline. Subsequently, the supernatant was discarded, and distilled water and PMSF (5 mM) were respectively added to the cell suspension. After five-time melting (at 37 °C) and freezing (at −80 °C), tachyzoites were crushed with a sonicator 15 times and each time sonicated for 10 sec (BANDELIN SONOPULS,GERMANY). The fragments of the parasite and cytoplasmic contents were used as total TLA, concentrated and then purified. Concentration and purification of the antiprotease, which interferes with cell culture, were performed by dialysis and by the addition of penicillin (100 IU/ml) and streptomycin (100 μg/m) through sterilization with a 0.22 filter. The concentration of soluble protein was determined by Bradford method, and the protein was kept at −80 °C until use (33, 34).

### Immunizations

For immunization experiments, we selected 45 BALB/c mice (6–8 wk old) and divided them into three groups of 15 mice. The mice were immunized with TLA alone or in combination with the TLR11 agonist as an adjuvant (AG-40B-0121).

The first group, considered as control, was injected with 100 μl of PBS. The second group was treated with 50 μl of TLA (20 μg/50 μl) and 50 μl of PBS injections. Finally, the third group was administered with 50 μm of TLA and 50 μl of adjuvant agonist TLR11 (1 μg/mouse). For immunization of mice, three injections, as vaccine boosters, were performed subcutaneously on the back of the neck on days 1, 10, and 20.

### MTT test for lymphocyte proliferation assay

Ten days after the last injection, five mice were selected from each group. The mice were necropsied, and spleens were isolated and homogenized in a homogenizer to separate the cells and then were centrifuged at about 450 ×g at room temperature for 5 min. The sediment was dissolved in 1 ml of RPMI 1640 (Gibco-BRL), and 15 ml of ammonium chloride (Sigma-Aldrich) 0.9% was added for the lysis of red blood cell. Then the sediment was centrifuged at 450 ×g at room temperature for 5 min, washed twice with RPMI medium and dissolved in 2 ml of complete RPMI containing 10% FBS (Gibco-BRL). Cell viability was measured using Trypan blue (Sigma-Aldrich). Spleen cells were cultured at the rate of 10^5^ cells/well in a volume of 100 μl of RPMI (containing 10% sterile FBS) in a 96-well plate. After cell culture, stimulant antigens containing 5 μl of *T. gondii* antigens (target group) and 95 μl of RPMI 1640 medium were added to the wells. Following a 48-hour incubation, 20 μl (5 mg/ml) of MTT ([3-(4,5-dimethylthiazol-2-yl)-2,5-diphenyltetrazolium bromide]) was added to each well and incubated at 37 °C for 4 h. Then the supernatant of the cells was discarded, and 100 μl of DMSO solution was added and dissolved with pipetting. After a 20-minute dissolving, the plate was read at 540 nm by an ELISA reader and compared between different groups (34,35).

### Cytokine assays

The levels of secreted INF-γ and IL-5 were estimated by using commercial ELISA kit (Mabtech AB, Nacka Strand, Sweden). Briefly, on the first day, ELISA plates were coated with mAb 1-D1K and incubated at 4–8 °C overnight. After an overnight incubation, the plates were washed twice with PBS, and the wells were blocked by an incubation buffer. After incubation at room temperature for 1 hour, the plates were washed five times with PBS containing 0.05% Tween 20. Subsequently, 100 μl/well of samples or standards were added and incubated at room temperature for 2 h, and washing was done as mentioned before. Next, 100 μl/well of mAb 7-B6-1-biotin was added and incubated at room temperature for 1 h. Washing was performed the same as the previous step, and 100 μl/well of streptavidin-HRP was added and incubated at room temperature for 1 h. Following washing step, 100 μl/well of an appropriate substrate solution was added. At the last stage, optical density at 450 nm in an ELISA reader was measured.

### Survival challenge

BALB/c mice were infected intraperitone-ally with 2 × 10^3^ tachyzoites of the *T. gondii* 10 d after the last immunization. Mortality was monitored daily for a period of 21 d after the challenge with strain RH (36).

### Statistical analysis

The results of MTT and cytokine levels measurements were analyzed using one-way analysis of variance (ANOVA) and Tukey’s post hoc test. The challenge test results were analyzed by using Kaplan-Meier test.

## Results

### Lymphocyte proliferation assay

Measurement of lymphocyte proliferation was carried out in both immunized and nonimmunized groups. We used the MTT method to evaluate the lymphocytic proliferation response to *T. gondii*. The mean OD in the PBS group was 0.181, and in the PBS + TLA and TLA + TLR11 groups, it was 0.293 and 0.236, respectively. A significant increase was observed in the proliferation of lymphocytes in mice receiving TLA + TLR11, as compared to the other groups.

### Cytokine evaluation assay

In the present study, INF-γ was evaluated as a marker for cellular immune response and IL-5 as a marker for humoral immune. Ten d after the last injection, supernatant of the mononuclear cell was evaluated for INF-γ and IL-5 in different mice groups. Evaluation of INF-γ and IL-5 secretion was conducted with an ELISA kit (Bender Med System, Vienna, Austria). The results showed a significant increase in the INF-γ titer in the TLA + TLR11 group (169.6 pg/ml) compared to the other two groups. In addition, a slight increase was observed in the IL-5 titer in mice receiving TLA + TLR11 adjuvant (48.4 pg/ml) in comparison to the other groups.

### Challenge test for survival time

**Fig. 1:**
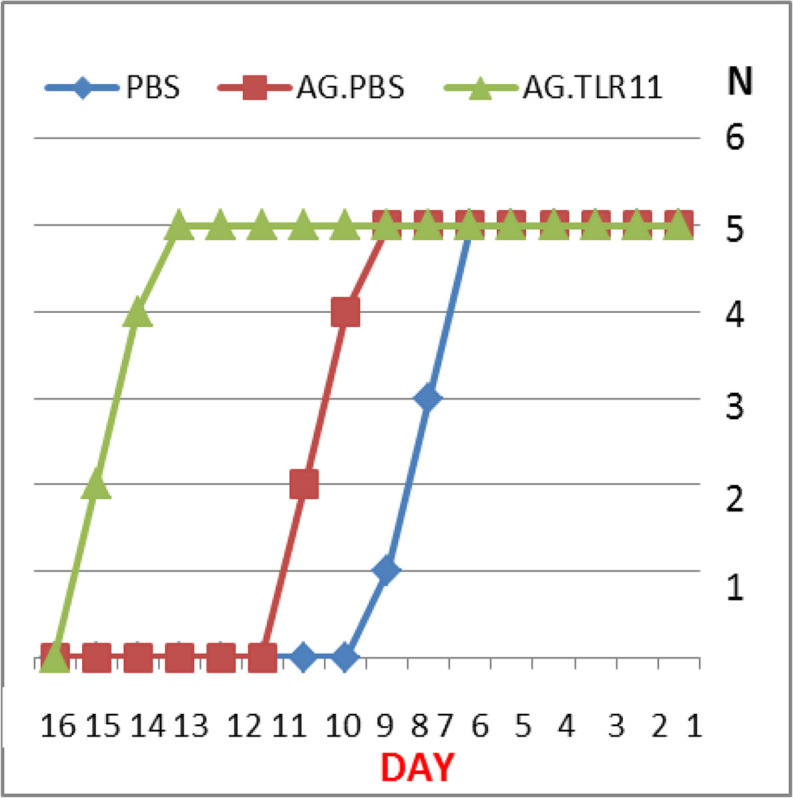
The percentage of survival time in mice immunized with TLA.TLR11

The Kaplan-Meier chart was drawn to evaluate the survival day in different groups of mice. The survival day significantly increased in mice received TLA+TLR11 adjuvant (15.2 d) in comparison to the other two groups (Fig. 1). The survival of mice receiving TLA receptor + PBS (10.2s d) was higher than that of mouse group that received PBS receptor alone (8.7s d).

## Discussion

Our results showed that the combination of TLR11 (as an adjuvant) and TLA significantly raised the effect of TLA by increasing the production of INF-γ and IL-5 and by the shift of the immune system to TH1. In addition, TLA + TLR11 adjuvant elevated the proliferation of lymphocytes and also the mice survival against *T. gondii*.

Adjuvants are commonly described as materials added to the formulations of vaccines to raise the immunogenicity of antigens (37). They help vaccine producers to use a smaller dose of antigen in each dose of vaccine (38). Adjuvants also affect the onset, strengh, and duration of immune responses (39). In addition to vaccine, adjuvants, as a supplement, have been used against *T. gondii* for a long time. Adjuvants are calssified into different categories, including mineral salt adjuvants (e.g., ALUM), tensoactive adjuvants (e.g., QuilA), bacteria-derived adjuvants (e.g., LPS), adjuvant emulsions (e.g., FCA), liposome adjuvants (probably better referred to as vesicular adjuvants to include NISV), polymeric microsphere adjuvants, carbohydrate adjuvants, and cytokine adjuvants (39).

Nowadays, multiple ingredients of *T. gondii* have been discovered to activate TLRs. The innate recognition of *T. gondii* via the TLR pathway has been well known. C57BL/6 mice infected i.p. with ME-49 strain of *T. gondii* have demonstrated that both TLR2 and TLR4 receptors may involve in the host defense against *T. gondii* infection when activated by the glycosylphosphatidylinositols and could work together with other MyD88-dependent receptors (40). Immature dentritic cells from C57BL/6 mice influenced by GM-CSF and IL-4 interact with *T. gondii* HSP-70 (agonist of TLR4) and induce protective immunity against this intracellular parasite (41). Intraperitoneal co-administration of Eimeria profilin-like protein with toxoplasma antigen in mice reduces brain cyst production up to 62% and increases the plasma level of IL-12p70. Therefore, toxoplasma antigen can induce both homoral and cellular immune responses. Based on the ratio of IgG2a to IgG1, when Toxoplasma antigen is injected with Eimeria profilin-like protein as an adjuvant, the shift of immune responses to Th1 is dominant. (42). Using CpGODN agonist of TLR9 (as an adjuvant) accompinead with TLA (as a vaccine) against *Toxoplasma* in genetically succeptable C57BL/6 mice led to an increase in INF-γ and toxoplasma-specific IgG and IgG_2a_ antibodies, suggesting a change in immune system shifted toward a Th1 pattern. Moreover, mice survival prolonged, and the brain cysts reduced by 64% (43).

The human TLR11 gene consists of some stop codons, and its expression in human does not perform for a full-length protein. However, our belief in TLR11 and other TLRs not expressed in human would provide us with prominent understanding of the pathogenesis of a variety of important human infections (2, 3, 13, 14). Investigation of TLR11 can contribute to promote our knowledge on human immune response against bacterial and apicomplexan pathogens carrying this kind of pathogen-associated molecular patterns because TLR11 regulates immune responses that share with other TLRs. In addition, TLR11-deficient mice are susceptible to human pathogens carrying this kind of PAMPs and may, therefore, be considered as wonderful animal models to examine corresponding human infectious diseases.

## Conclusion

Profilin (as an adjuvant) in combination with TLA could be a potent vaccine candidate that evokes a powerful specific immune response and significantly improves the efficacy of TLA vaccine by increasing the induction of INF-γ production and by shifting the immune responses to Th1 profile through increasing the INF-γ/IL-5 ratio. It causes significant protection against *T. gondii* after i.p. injection.
